# SLC6A1 Neurodevelopmental Disorder: A Case Report

**DOI:** 10.1192/bjo.2025.10757

**Published:** 2025-06-20

**Authors:** Nikita Pillay, Gurjot Brar, Niamh Mulryan

**Affiliations:** AVISTA, Dublin, Ireland

## Abstract

**Aims:** Solute Carrier Family 6 member 1 (SLC6A1) genetic mutations are rare and mostly reported in the paediatric population. Few reports on adult cases have been mentioned in the literature. At present only pathological and likely pathological variants can confirm a diagnosis of SLC6A1-related neurodevelopmental disorder.

**Methods:** We report the clinical presentation of an adult male with a heterozygous variant of uncertain significance of the SLC6A1 gene. He presented with developmental delay initially and only in later years presented with other features in keeping with SLC6A1-related neurodevelopmental disorder. This includes moderate intellectual disability, epilepsy, autism, attention deficit disorder, behavioural difficulties including aggression. A further EEG at age 14 also showed focal and general abnormalities, however an MRI was normal. He was trialled on methylphenidate, lis-dexamphetamine and atomoxetine which were all unsuccessful. In 2024, genetic testing revealed a heterozygous variant of uncertain significance of the SLC6A1 gene.

**Results:** Our case adds further credence to the growing literature on SLC6A1 gene-related disorder and SLC6A1-related neurodevelopmental disorder. This individual illustrates the diverse clinical phenotype. Given the lack of published evidence, the result from genetic testing for this person was classified as a variant of uncertain significance.

Uniquely, this case report describes an adult who has been tested, as opposed to paediatric cases which comprise much of the literature in this area. The notable case described by Verhowen of a patient who presented with a late onset of seizures, similar symptoms, progression and prior failed trials of medication as the case presented here, also benefited from genetic testing in later life. In addition, this patient responded well to sodium valproate which is known to be beneficial in cases with SLC6A1. This is most likely related to the GABA mediation. The findings of this case demonstrate the SLC6A1 gene-related disorder provides a unifying explanation for this diverse clinical phenotype, previously thought to be a constellation of syndromes and co-morbid symptoms.

The individual in this case study failed to respond to three adequate trials of medication for ADHD, all which have a robust evidence base. This suggests a possible alternate pathway for the development of ADHD features in cases such as this, as the majority of individuals with moderate-severe ADHD symptoms achieve symptomatic relief with pharmacological intervention

**Conclusion:** This case highlights the relevance of genetic testing in adults and the reporting of variants of uncertain significance that could possibly lead to reclassification of a variant.

